# Adalimumab-Induced Hepatocellular Injury in a Young Male With Hidradenitis Suppurativa and Underlying Metabolically Dysfunctional Liver Disease: A Case Report

**DOI:** 10.7759/cureus.100354

**Published:** 2025-12-29

**Authors:** Nada M Abdulhameed, Mohammed Banama, Esmaeel Alsayed AlMarzooqi

**Affiliations:** 1 Otolaryngology - Head and Neck Surgery, Graduate Medical Education, Mohammed Bin Rashid University of Medicine and Health Sciences, Dubai, ARE; 2 Internal Medicine, Mohammed Bin Rashid University of Medicine and Health Sciences, Dubai, ARE; 3 Gastroenterology and Hepatology, Dubai Health, Dubai, ARE; 4 Dermatology, Mohammed Bin Rashid University of Medicine and Health Sciences, Dubai, ARE; 5 Dermatology, Dubai Health, Dubai, ARE

**Keywords:** adalimumab (humira), anti-adalimumab antibodies, biologic therapy, drug-induced liver injury (dili), hidradenitis suppurativa (hs), metabolic dysfunction-associated steatotic liver disease (masld), tumor necrosis factor-alpha (tnf-α) inhibitors

## Abstract

Adalimumab is widely used for the management of moderate-to-severe hidradenitis suppurativa (HS). We report the case of a 20-year-old male with Hurley stage 2-3 HS who developed progressively rising liver enzymes shortly after initiating adalimumab therapy. His alanine aminotransferase (ALT) level was 40 U/L at baseline, increased to 67 U/L one month after treatment, and continued to rise over the following year, reaching a peak of 139 U/L. Autoimmune serologies, ceruloplasmin, and viral hepatitis testing were all negative, effectively excluding autoimmune and viral etiologies. Abdominal ultrasound with elastography demonstrated grade 3 hepatic steatosis without fibrosis, consistent with underlying metabolic dysfunction-associated steatotic liver disease (MASLD). The isolated hepatocellular pattern of enzyme elevation, combined with the temporal association with adalimumab initiation and the exclusion of alternative causes, supported a diagnosis of adalimumab-associated drug-induced liver injury occurring on a background of MASLD. Adalimumab was discontinued, and the patient was counseled to begin structured lifestyle modification. This case highlights the importance of vigilant hepatic monitoring in patients receiving anti-TNF-α therapy, particularly those with metabolic risk factors that may increase susceptibility to liver injury.

## Introduction

Tumor necrosis factor-alpha (TNF-α) is the therapeutic target of adalimumab, a fully human monoclonal antibody used to reduce downstream inflammatory cytokine signaling. Adalimumab is approved for several inflammatory conditions, including rheumatoid arthritis, psoriatic arthritis, ankylosing spondylitis, psoriasis, and Crohn’s disease [1]. The most common adverse effect is injection-site reactions, occurring in more than 10% of treated patients, while serious risks include opportunistic infections such as tuberculosis reactivation. Less frequently, adalimumab has been associated with systemic autoimmune phenomena, demyelinating disease, and lymphoma [2].

Hidradenitis suppurativa (HS) is a chronic inflammatory skin disorder characterized by recurrent nodules, abscesses, and scarring, often requiring systemic therapy. Adalimumab was the first FDA-approved biologic treatment for moderate-to-severe HS and has demonstrated significant improvements in disease severity and quality of life, with pivotal phase 3 trials (PIONEER I and II) showing that approximately 50% of patients achieve Hidradenitis Suppurativa Clinical Response (HiSCR) with weekly therapy [3]. However, although generally well tolerated, adalimumab has been associated with uncommon hepatic adverse effects, including autoimmune-like hepatitis and hepatocellular injury [4].

In recent years, newer biologic agents, particularly those targeting the IL-17 pathway, have demonstrated substantially higher response rates, with phase 3 trials of secukinumab, bimekizumab, and brodalumab reporting HiSCR achievement in 70-80% of patients [5-7]. As additional therapies continue to emerge, the treatment landscape for HS is evolving, with these newer biologics offering potentially superior efficacy compared to adalimumab.

At the same time, metabolic dysfunction-associated steatotic liver disease (MASLD) is becoming increasingly prevalent in young adults. MASLD commonly presents with mild ALT elevation and can complicate the evaluation of abnormal liver enzymes in patients receiving biologic therapy. Because MASLD increases hepatic susceptibility through mechanisms such as oxidative stress and impaired mitochondrial resilience, distinguishing underlying metabolic liver disease from drug-induced liver injury (DILI) is crucial when interpreting liver function abnormalities in patients treated with TNF-α inhibitors [8]. Accurate differentiation between these entities is essential to ensure patient safety and guide appropriate therapeutic decisions.

## Case presentation

A 20-year-old man with a four-year history of hidradenitis suppurativa involving the bilateral axillae and groin was evaluated for progressively rising liver enzymes. His disease was initially classified as Hurley stage 3 but was later reclassified as stage 2. He had initially been prescribed lymecycline and topical clindamycin, but did not adhere to these treatments. Adalimumab therapy was subsequently initiated, beginning with a 160 mg loading dose, which he tolerated well, followed two weeks later by the maintenance regimen of 80 mg every two weeks. He reported good tolerance to treatment and noted a reduction in active lesions.

**Figure 1:**
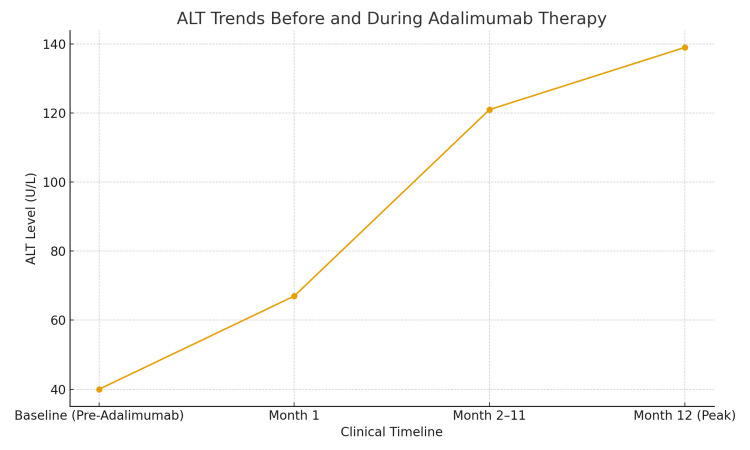
ALT trends before and during adalimumab therapy. ALT: alanine aminotransferase

Baseline laboratory testing showed an ALT of 40 U/L. One month after starting adalimumab, his ALT increased to 67 U/L and continued to rise over the following year, reaching a peak of 139 U/L (Figure 1). He denied alcohol consumption, smoking, illicit drug use, or intake of hepatotoxic supplements. He also reported no symptoms of jaundice, nausea, vomiting, or abdominal pain. There was no family history of liver disease and no significant medical or surgical history.

Viral hepatitis screening, including HBsAg, HBcIgM, HCV antibody, and HIV Ag/Ab, was negative. Autoimmune workup, including anti-nuclear antibody (ANA) and anti-liver kidney microsomal antibodies, was also negative, and IgG levels were normal. Albumin, bilirubin, and INR remained within normal limits. The isolated hepatocellular alanine aminotransferase (ALT) elevation occurring after the initiation of adalimumab suggested DILI in the setting of underlying MASLD, after viral, autoimmune, and obstructive causes had been excluded.

**Figure 2:**
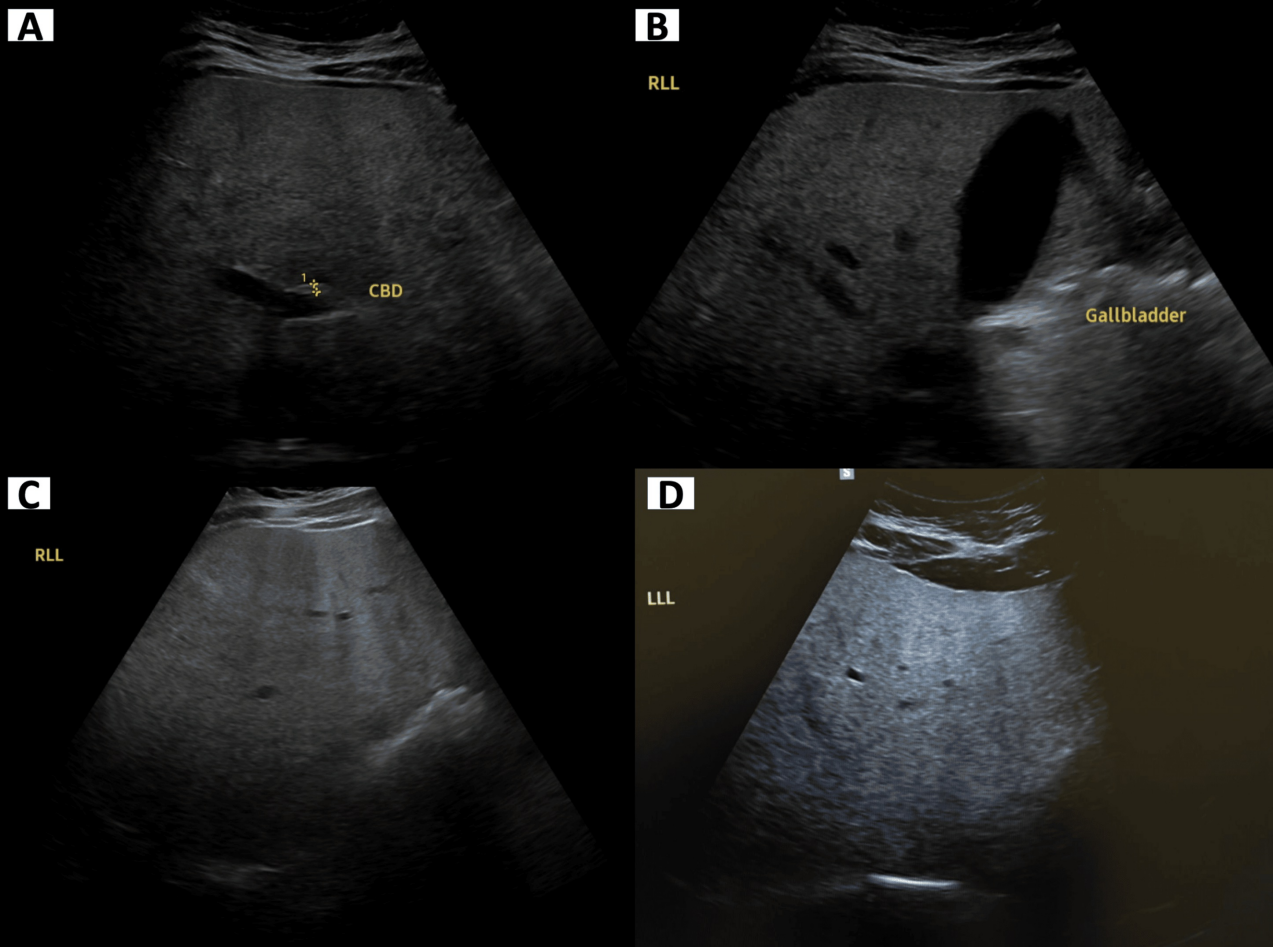
Abdominal ultrasound demonstrating diffusely increased hepatic echogenicity consistent with hepatic steatosis. (A) Common bile duct (CBD) with normal caliber. (B) Gallbladder with no evidence of cholelithiasis or cholecystitis. (C) Right lobe of the liver demonstrating increased parenchymal echogenicity consistent with diffuse hepatic steatosis. (D) Left lobe of the liver showing increased echogenicity consistent with fatty infiltration.

An abdominal ultrasound with elastography was subsequently performed, which demonstrated hepatomegaly with increased hepatic echogenicity consistent with grade 3 steatosis, without evidence of fibrosis (Figure 2).

Based on the combined laboratory and imaging findings, the patient was advised to discontinue adalimumab and begin structured lifestyle modification, including weight reduction and 300 minutes per week of moderate-intensity exercise. Repeat liver function testing was planned following cessation of adalimumab.

## Discussion

Adalimumab has been associated with rare hepatotoxic reactions, including idiosyncratic hepatocellular injury and autoimmune-like hepatitis. Most reported cases occur within the first one to three months of therapy and typically present with elevated transaminases and normal bilirubin levels [9], findings consistent with the early pattern observed in our patient. However, the presence of grade 3 steatosis on imaging suggests that underlying MASLD likely contributed to increased hepatic susceptibility.

This case represents a likely example of adalimumab-induced hepatocellular injury occurring on a background of MASLD. Several factors support this interpretation. First, there was a clear temporal association, with normal baseline ALT, followed by a progressive and sustained rise after initiation of adalimumab, a pattern characteristic of unpredictable biologic-related DILI. Second, a comprehensive exclusion of alternative etiologies was performed, including negative viral hepatitis markers, negative autoimmune serology, normal ceruloplasmin, and abdominal imaging without obstructive pathology, which strengthens the causal link to adalimumab. Third, the presence of grade 3 steatosis without fibrosis indicates significant underlying metabolic liver disease. Patients with MASLD commonly exhibit reduced hepatic reserve, increased oxidative stress, and heightened vulnerability to drug-induced hepatotoxicity, which likely amplified the risk of liver injury in this patient.

Drug-induced liver injury accounts for nearly 50% of acute liver failure cases and approximately 10% of acute hepatitis presentations [10]. Hepatotoxicity may occur through either direct drug toxicity or immune-mediated mechanisms. These reactions are typically unpredictable, dose-independent, vary widely in severity, and may occur in patients without identifiable risk factors. Because of this variability, it is not possible to determine in advance which individuals will develop an adverse drug reaction. Although cholestatic injury represents the most common pattern of DILI, hepatocellular injury, as seen with adalimumab, can also occur. Importantly, hepatic enzyme abnormalities may persist even after the offending agent is discontinued, and in rare cases, a self-perpetuating autoimmune-like process may lead to chronic liver injury [11].

MASLD has emerged as the most frequent cause of elevated ALT in young adults. Through mechanisms such as increased oxidative stress, impaired mitochondrial function, and altered cytokine signaling, MASLD enhances hepatocyte vulnerability and may potentiate drug-induced injury [12]. In this case, the coexistence of significant steatosis and a temporal association between adalimumab initiation and rising ALT levels strongly supported a diagnosis of adalimumab-associated DILI superimposed on MASLD. The isolated hepatocellular pattern with preserved bilirubin and synthetic function further supports this diagnosis.

For patients with metabolic risk factors, early recognition of abnormal liver enzyme trends and prompt discontinuation of the suspected agent are essential to preventing progression to more severe liver injury. This case highlights the importance of close hepatic monitoring during biologic therapy, particularly in individuals with underlying MASLD.

In this patient, the diagnosis of adalimumab-induced hepatocellular injury superimposed on MASLD was supported by the clear temporal relationship between initiation of adalimumab and rising ALT levels, along with the exclusion of viral, autoimmune, or cholestatic etiologies. Taken together, these findings strongly favor a drug-induced mechanism rather than primary liver disease alone. The patient’s progressive hepatocellular enzyme elevation is most consistent with adalimumab-induced liver injury, with significant underlying MASLD acting as a major susceptibility factor. Discontinuation of adalimumab and aggressive metabolic risk reduction represent the safest management approach, and rechallenge is not recommended. These findings reinforce the need for heightened vigilance when prescribing TNF-α inhibitors to patients with MASLD, as timely recognition and cessation of the offending medication are critical to preventing more significant hepatic damage.

## Conclusions

Although adalimumab-induced liver injury is rare, any elevation in transaminases following treatment initiation warrants prompt evaluation. The coexistence of MASLD may increase susceptibility to hepatic injury and complicate the interpretation of liver enzyme abnormalities. This case highlights the importance of early recognition of abnormal trends, timely discontinuation of the suspected agent, and routine hepatic monitoring during biologic therapy. Ongoing management of MASLD, including weight reduction and control of metabolic risk factors, remains essential, particularly when patients are receiving medications with potential hepatotoxicity.
